# Oncogenic Chromatin Modifier KAT2A Activates MCT1 to Drive the Glycolytic Process and Tumor Progression in Renal Cell Carcinoma

**DOI:** 10.3389/fcell.2021.690796

**Published:** 2021-06-29

**Authors:** Yuanyuan Guo, Beibei Liu, Yihan Liu, Wei Sun, Wuyue Gao, Shilong Mao, Li Chen

**Affiliations:** ^1^Department of Urology, The First Affiliated Hospital of Bengbu Medical College, Anhui, China; ^2^Department of Epidemiology and Biostatistics, School of Public Health, Nanjing Medical University, Nanjing, China; ^3^Department of Pharmacy, Shanghai Xuhui District Central Hospital, Xuhui Hospital of Zhongshan Hospital, Fudan University, Shanghai, China

**Keywords:** KAT2A, chromatin modifier, MCT1, glycolysis, renal cell carcinoma

## Abstract

**Objectives:**

This study aims to investigate the underlying mechanisms of KAT2A/MCT1 axis in renal cell carcinoma (RCC), providing potential therapeutic targets.

**Methods:**

We obtained the expression data of KAT2A and MCT1 from The Cancer Genome Atlas Kidney Clear Cell Carcinoma (TCGA-KIRC) and International Cancer Genome Consortium (ICGC) databases. Differential analysis was conducted via the limma package. The CCK8 assay, soft agar assay, clone formation assay, and patients-derived organoid models were used to detect cell growth. The transwell and wound-healing assays were utilized to detect cell migration. The *in vitro* and *in vivo* assays were further conducted to assess the oncogenic roles of KAT2A. The transcriptome sequencing and chromatin immunoprecipitation (ChIP) sequencing were conducted to screen KAT2A downstream targets. The dose-effect curves were used to detect the 50% inhibiting concentration (IC50) of AZD3965. Data analysis was performed in the Graphpad Prism (Version 8.3.0) and R software (Version 3.6.1).

**Results:**

Our study found that KAT2A was highly expressed in RCC versus normal samples. Prognostic analysis indicated that a high KAT2A was an independent biomarker and associated with poor survival outcomes. KAT2A could promote RCC proliferation and distal metastasis *in vitro* and *in vivo*. Transcriptome analysis and ChIP-seq were combined to find that KAT2A mainly regulated the glycolytic process. Validation and rescue assays revealed that MCT1 was the downstream target of KAT2A, and KAT2A depended on MCT1 to promote RCC malignant phenotypes. Lastly, MCT1 inhibitor (AZD3965) was effective to treat KAT2A-induced RCC progression.

**Conclusion:**

Our study indicated that KAT2A was an oncogenic chromatin modifier that promotes RCC progression by inducing MCT1 expression. We proposed that MCT1 inhibitor (AZD3965) was useful for suppressing RCC.

## Introduction

Renal cell carcinoma (RCC) belongs to one of the most common urological malignancies, accounting for ∼2–3% of all malignant diseases worldwide (Saad et al., 2019; Siegel et al., 2021). Laparoscopic surgery was the mainstream strategy to treat localized renal tumors, whereas there were few effective methods that could be used for metastatic cases (Wiechno et al., 2018). Meanwhile, kidney cancer exhibits a natural resistance to chemotherapy or radiotherapy (Barata and Rini, 2017). Currently, there are several molecular targeting drugs, including inhibitors targeting vascular endothelial growth factor (such as sunitinib, sorafenib, bevacizumab) or mammalian target of rapamycin (everolimus) (Choueiri and Kaelin, 2020). However, these drugs exhibited a limited efficacy in a subset of patients and safety needs to be further evaluated. As a result, novel biomarkers and strategies are still warranted for RCC treatment.

Aberrant epigenetic remodeling represents a molecular hallmark that contributes to tumorigenesis and progression (Hou et al., 2020; Nacev et al., 2020; Okazaki et al., 2020). High-throughput sequencing data across RCC tumors have identified several pivotal epigenetic drivers with a high mutated frequency, including VHL, SETD2, BAP1, and PBRM1 (Sato et al., 2013; Dizman et al., 2020; Wang X. M. et al., 2020). Whether there existed other prognostic or therapeutic epigenetic targets remained interesting to be investigated further. KAT2A, also known as Gcn5, belongs to a histone acetyltransferase that transfers acetyl groups to the lysine residues which mediates the post-translational modification at various positions of histone H3 (Wang et al., 2017). Moreover, the succinyl-coenzyme A (succinyl-CoA) is found to bind to KAT2A, which could also function as a succinyltransferase and succinylates histone H3 on lysine 79 (Wang et al., 2018). The succinyltransferase activity of KAT2A is essential for histone succinylation and indispensable for tumor growth (Tong et al., 2020). Previous studies have indicated that KAT2A is an essential candidate, and targeting KAT2A could significantly induce myeloid differentiation and apoptosis to suppress the tumor growth of acute myeloid leukemia (AML) (Tzelepis et al., 2016). In addition, KAT2A-mediated histone succinylation regulates β-catenin stability to mediate the progression of pancreatic ductal adenocarcinoma (PDAC) (Tong et al., 2020). In our previous studies, we observed that KAT2A was significantly elevated in RCC. However, the specific roles of KAT2A in RCC have never been reported and remained indefinite.

Metabolic reprogramming of RCC has a significant impact on the tumor progression and distal metastasis, which shed light on the alternative strategies for the treatment of RCC (Wettersten et al., 2017; Green et al., 2019; Nishida et al., 2020). Warburg effect is a common biological phenomenon in RCC which refers to the cancer cells that preferentially depend on aerobic glycolysis to obtain energy, although with an oxygen-rich environment (Lu et al., 2015; Nakagawa et al., 2020; Shi et al., 2020). However, the underlying mechanisms of dysregulated glucose metabolism still remained unclear. The monocarboxylate transporters (MCTs), especially MCT1 and MCT4, are important contributors to induce enhanced glycolysis and tumor aggressiveness (Benjamin et al., 2018; Tasdogan et al., 2020; Zhao et al., 2020). Particularly, researchers have already developed effective MCT1 inhibitors to suppress tumor growth, including small cell lung cancer (Polański et al., 2014), leukemia, or neuroblastoma. It still remains indefinite whether MCT1 could mediate glycolysis remodeling and serve as an effective target in RCC.

In the current study, we discovered the oncogenic role of KAT2A in RCC and described the underlying mechanisms between KAT2A and MCT1, proposing that KAT2A/MCT1 axis may be a novel predictive biomarker and therapeutic target for RCC.

## Materials and Methods

### Cell Lines and Culture

The human RCC cell lines (Caki-2, SLR-21, 786-O, and A498) and 293 T were derived from the American Type Culture Collection (ATCC). The cells were maintained in RPMI-1640 (Gibco, Carlsbad, CA, United States) containing 10% FBS (Gibco, Carlsbad, CA, United States) and 100 μg/ml of streptomycin under 37°C with 5% CO_2_°According to the expression data in the CCLE dataset, Caki-2, SLR-21, and A498 are divided into the KAT2A^high^ cells, whereas 786-O belongs to the KAT2A^low^ cells.

### Patient Samples

The 15 pairs of RCC tumor samples and matched normal kidney tissues were obtained from the department of urology in the first affiliated hospital of Bengbu medical college from July 2017 to September 2020. The tissues were frozen and maintained in liquid nitrogen for following analysis. Besides, FFPE samples of RCC and the corresponding normal samples were collected upon resection and stored in formalin. The protein levels of genes in fresh RCC samples were determined via Western blotting. Meanwhile, the clinical parameters of RCC were further collected correspondingly including TNM stages, tumor grades, pathological subtypes, age, and gender. The study was reviewed and approved by the Ethics Committee of the first affiliated hospital of Bengbu Medical College, and informed consents were all written by the RCC patients in this study.

### Generation of Stable *CRISPR-Cas9* Mediated KAT2A-Knockout Cells

The guide oligos (sgRNAs) targeting KAT2A gene were designed and cloned into the pX459 plasmid. RCC cell lines (A498 and Caki-2) were plated and transfected pX459 constructs for 24 h. Then, 1 μg/ml of puromycin was added to screen cells for 3 days. The monoclonal cell line was derived when seeding the living cells into 96-well plates with limited dilution. Western blot assays and corresponding sanger sequencing were utilized to confirm the knockout efficiency of genes. The specific sgRNAs targeting KAT2A were summarized as follows: sgRNA (KAT2A-KO#1) F: 5-CACCGCCTGAGACCTCTGCCGAAAC-3; R: 5-AAACGTTTCGGCAGAGGTCTCAGGC-3; sgRNA (KAT2A-KO#2) F: 5-CACCGCCTACAAGGTCAATTACACC-3; R: 5-AAACGGTGTAATTGACCTTGTAGGC-3.

### Clone Formation Assay and 3D Soft Agar Assay

Cells (400 cells per well) were seeded into a 6-well plate and incubated for 2 weeks until cell clones appeared. Then, the colonies of cells were fixed with methanol and stained with Giemsa. Cell counting kit-8 (CCK-8) assay (MedChemExpress, United States) was used to determine the short-term cell proliferation. Briefly, the cells were seeded onto the 96-well plates (1,000 cells/well). A total of 10 μl of the CCK-8 solution was added into the culture and incubated for 2 h. The optical density was assessed using a microplate reader at 450 nm. Each assay was carried out in triplicate. To conduct the soft agar colony formation experiment, 10% FBS and 0.7% agar were contained with 2 ml of gel. The cells were then seeded into the medium including 10% FBS with 0.35% agar and incubated at 37°C for 21 days with the density of 1 × 10^5^ cells per well. The number of soft agar colonies was scored and compared by the ImageJ software.

### Migration and Invasion Assay

Caki-2 and A498 cells were maintained in a serum-free medium for 2 days. For the migration assay, a total of 3 × 10^4^ cells were seeded in a serum-free medium in the upper chamber (8 μm pore size; Corning, Beijing, China). Besides, the lower chamber was filled with RPMI1640 containing 5% FBS. The cells were then fixed in 4% paraformaldehyde for 15 min and stained with crystal violet after 48 h. For the invasion assay, the membranes were covered with Matrigel (BD Biosciences). The migrated cells were stained and counted in nine different fields.

### Lentiviral Preparation, Viral Infection, and Stable Cell Generation

We purchased the pLKO.3G GFP-shRNA plasmids from Addgene. After 48 h of transfection, the viruses were collected from the medium. Cells were infected with the collected viruses over 2 days with the company of polybrene, which were then sorted via GFP signals for 4 days. The specific shRNA sequences were listed as follows: KAT2A-shRNA#1 Sense: 5-GATCCGTACCGGAGACACCAAGCAGGTCTATTTCTCGAG AAATAGACCTGCTTGGTGTCTTTTTTTGG-3; Anti-sense: 5-AATTCCAAAAAAAGACACCAAGCAGGTCTATTTCTCGAG AAATAGACCTGCTTGGTGTCTCCGGTACG-3. KAT2A-shR NA#2 Sense: 5-GATCCCCGGGCTGAACTTTGTGCAGTACAA CTCGAGTTGTACTGCACAAAGTTCAGCTTTTTGG-3; Anti -sense: 5-AATTCCAAAAAGCTGAACTTTGTGCAGTACAAC TCGAGTTGTACTGCACAAAGTTCAGCCCGGG-3. MCT1- shRNA#1 Sense: 5-GATCCCCGGGCTCCGTATTGTTTGA AACATCTCGAGATGTTTCAAACAATACGGAGCTTTTTGG-3; Anti-sense: 5-AATTCCAAAAAGCTCCGTATTGTTTGA  A A C A T CTCGAGATGTTTCAAACAATACGGAGCCCGGG-3; MCT1-shRNA#2 Sense: 5-GATCCCCGGTATGTTTCTGCTAG CTATATACTC G A G T A TATAGCTAGCAGAAACATATTTTTG G-3; Anti-sense: 5-AATTCCAAAAATATGTTTCTGCTAGCTA TATACTCGAGTATATAGCTAGCAGAAACATACCGGG-3.

### Western Blot Assay

The cells were collected and lysed in the lysis buffer which was mixed with 0.1% sodium dodecyl sulfate (SDS), 50 mM TrisHCl, sodium deoxycholate, 150 mM NaCl, 1% NP-40, and a protease inhibitor (PH = 7.5). Then, the protein samples were loaded to 8% sodium dodecyl sulfate-polyacrylamide gel electrophoresis (SDS-PAGE) and transferred to a polyvinylidene difluoride membrane (PVDF). After blocking with 5% non-fat dry milk for 1 h, the membranes were incubated with the specific primary antibody overnight. After washing in TBST for three times in 15 min, the membranes were then incubated with the secondary antibodies (peroxidase-labeled anti-mouse and anti-rabbit antibodies) for 1 h. Briefly, the primary antibodies were utilized at a dilution of 1:2,000, while secondary antibodies were diluted with 1:5,000. After washing in TBST for three times in 15 min, the membranes were finally visualized in an ECL + plus^TM^ Western Blotting system kit (cat. no. RPN2232; Amersham). The detailed information of the primary antibodies was summarized as follows: KAT2A (abcam, ab217876); MCT1 (abcam, ab90582); β-Actin (CST#4970).

### RNA-Seq, Chromatin Immunoprecipitation (ChIP)-Seq Assay, and ChIP-qPCR

Total RNA from Caki-2 cells were extracted from with or without KAT2A deficiency, which were then subjected to HiSeq RNA-Seq based on the Jiayin Tech Solutions Co platform. Each RNA sample was designed to three biological replicas and was finally mixed to analyze for further minimizing the variations among samples. The genome (hg19) based on the Bowtie tool was utilized to map the final transcriptome reads. The gene expression data was further normalized by the RSEM software. For the ChIP-seq experiment, the Caki-2 cells were cross-lined and lysed, and sonication was used to shear the DNA fragments into 200–800 bp. The ChIP-seq assays were conducted via Active Motif using the KAT2A antibody (abcam, ab217876). The nucleotide reads derived from the Illumina sequencing were mapped on the genome through the BWA algorithm, and the average profile for peak distributions was generated via deeptools. For the ChIP-qPCR assays, Quantitative PCR analysis was conducted following the ChIP experiment. Relative occupancy of the promoter region of MCT1 and CCND1 was normalized to the normal rabbit IgG.

### Immunohistochemistry (IHC)

In total, 15 paired RCC samples and paired normal tissues were collected from the first affiliated hospital of Bengbu Medical College via laparoscopic surgery. The formalin-fixed paraffin-embedded (FFPE) tissues and hematoxylin and eosin (H&E) slides were confirmed by a pathologist. Then, the samples were incubated with H_2_O_2_ (15%) to suppress the endogenous peroxidase activity for 15 min under room temperature. The slides were blocked using the normal goat serum for 1 h and incubated with primary antibody (KAT2A, ab217876) overnight. After washing three times in 1 × PBS, the sections were incubated with biotinylated goat-anti–rabbit IgG secondary antibodies for 30 min. After washing three times in 1 × PBS, the samples were further incubated with HRP. After washing three times in 1 × PBS for 15 min, specific samples were finally developed with diaminobenzidine. Lastly, the IHC staining of KAT2A was scored and analyzed by two independent pathologists.

### Animal Xenografts Study

The 4–6-week-old BALB/c nu/nu mice were purchased from Mu Tu Biological Technology (Nanjing, China), which were maintained in our institutional pathogen-free mouse facilities. Briefly, 6 × 10^6^ indicated A498 and Caki-2 RCC cells were suspended in 150 μl of PBS buffer and injected into the flanks of male nude mice. Tumor growth of mice was monitored every 2 days, for a total period of 30 days. At the end of 4 weeks, all mice were sacrificed, and *in vivo* solid tumors were dissected and analyzed. All experimental protocols were reviewed and approved by the Ethics Review Committee for Animal Experimentation of Bengbu medical college.

### Bioinformatics Analysis

The expression data of RCC patients were obtained and collected from the public dataset, including TCGA-KIRC^1^, ICGC-RCC^2^, and GSE40435^3^. The differential analysis was performed via the limma package. The univariate Cox regression analysis and Kaplan-Meier analysis were mainly conducted by the survival package. The enriched biological items were obtained via the clusterprofiler package. The ATAC-seq data in RCC was obtained and analyzed from the TCGA dataset^4^.

### Statistical Analysis

All data derived in this study were shown as Mean ± SD for assays which were conducted with at least three replicates. The Student’s *t*-test was used to compare the difference between two groups. The log-rank test was used to analyze the Kaplan-Meier curves. Spearman’s rank correlation analysis was conducted to analyze the associations between KAT2A and clinical parameters. All data analysis was performed in the R studio (Version 3.6.0) and Graphpad prism (Version 8.0). The *P-*values < 0.05 were considered to be statistically significant.

## Results

### KAT2A Was Highly Expressed in Kidney Cancer and Has Prognostic Significance

**FIGURE 1:**
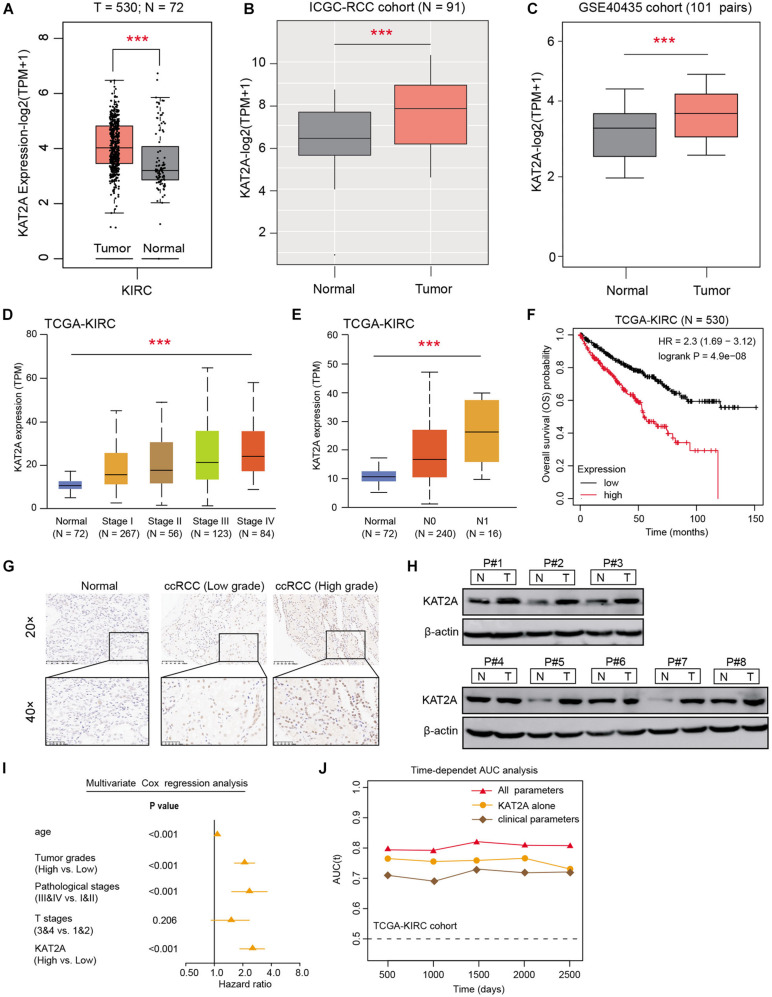
High KAT2A was an independent risk factor in renal cell carcinoma. **(A–C)** Public data analysis revealed that KAT2A was significantly higher in the RCC tumor samples versus normal tissues in the TCGA-KIRC cohort, ICGC-RCC cohort, and GSE40435 dataset. **(D,E)** Correlation analysis suggested that KAT2A expression levels were positively associated with advanced pathological stages (*P* < 0.0001) and lymph nodes metastasis (*P* < 0.0001). **(F)** Kaplan-Meier analysis indicated that patients with high KAT2A levels suffered from worse survival outcomes versus those with low KAT2A levels (Log-rank test *P* < 0.0001). **(G)** Representative immunohistochemical staining for KAT2A in RCC (low-grade and high-grade) and adjacent normal kidney tissues. **(H)** The protein levels of KAT2A were further assessed in RCC fresh tissues and matched normal kidney tissues through Western blotting (*N* = 8 pairs). **(I)** Multivariable analyses were conducted in the RCC cohort and illustrated by forest plot. All bars correspond to 95% CIs. **(J)** The time-dependent receiver operating characteristic (ROC) analysis for KAT2A risk scores, clinical parameters, and the combined all variables. ***P < 0.001.

Previous studies have already indicated that aberrant KAT2A was associated with aggressive phenotypes across various tumors, however, its specific effect in kidney cancer still remains unclear. To investigate the role of KAT2A in RCC, we collected the expression data of RCC samples from the TCGA-KIRC cohort. We found that KAT2A was significantly higher in 530 tumor tissues than 72 normal samples with *P* < 0.001 (Figure 1A). Besides, we also observed the elevated expression levels of KAT2A in tumor tissues across other RCC cohorts, including the ICGC-RCC cohort (Figure 1B) and GSE40435 (Figure 1C). Correlation analysis was then conducted and suggested that KAT2A expression levels were positively associated with the clinical risk factors based on the TCGA-KIRC cohort, including pathological stages, tumor grades, or TNM stages (Figures 1D,E and Supplementary Figure 1A). In addition, we classified the TCGA-KIRC patients into high-level and low-level groups with the median data of KAT2A expression as the cutoff, and Kaplan-Meier analysis suggested that patients with a high KAT2A suffered from worse survival outcomes than patients with a low KAT2A (Log-rank test *P* < 0.001, Figure 1F). We confirmed these findings via immunohistochemistry (IHC) in the RCC tumor samples from our hospital, in which immunostaining scores of KAT2A was notably higher in the advanced tumor samples than low-grade tumors or normal tissues (Figure 1G). In line with the above results, the KAT2A protein levels were found to be notably higher in 6/8 (75%) of the human fresh RCC tissues than paired normal kidney tissues via Western blotting (Figure 1H). Lastly, multivariate Cox regression analysis was conducted to suggest that KAT2A expression level, age, pathological stages, tumor grades, and T stage were all independent factors and associated with a 5-year survival of RCC patients (Figure 1I). Particularly, KAT2A was an independent predictive marker for the prognosis of RCC patients [HR = 2.824, 95% CI (1.873–3.816)] and the 5-year AUC of the ROC curve was 0.824 (Supplementary Figure 1B). The time-dependent receiver operating characteristics (ROC) curve analysis was conducted to suggest that the integration of KAT2A risk scores and clinical risk factors exhibited much more superior than either one alone in the RCC cohort (Figure 1J). The clinical baseline of RCC patients were summarized in Supplementary Table 1.

### High KAT2A Promotes RCC Tumor Growth *in vitro* and *in vivo*

**FIGURE 2:**
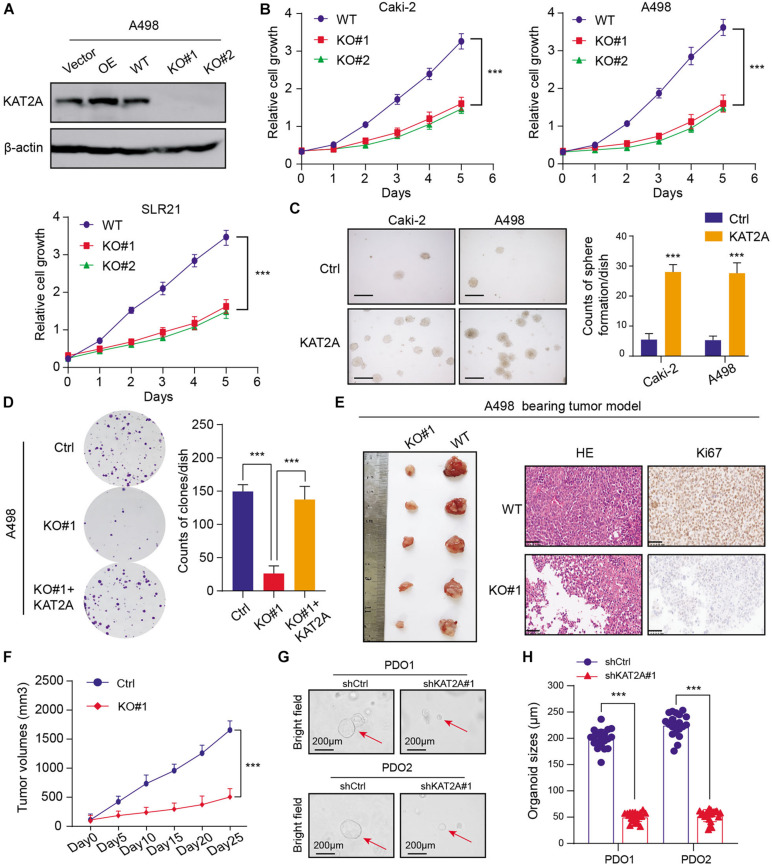
Functional assays validated that KAT2A promoted proliferation of RCC *in vitro* and *in vivo*. **(A)** The protein levels of KAT2A in A498 cells with KAT2A overexpression or deficiency were validated via Western blotting. **(B)** CCK-8 assays were performed to reveal that KAT2A deficiency could significantly suppress the *in vitro* growth of RCC cells, including Caki-2, A498, and SLR21. **(C)** KAT2A overexpression could significantly promote the growth of RCC cells revealed by the 3D soft-agar colony formation assay. **(D)** The clonogenic efficiency in A498 cells with wild type or deleted KAT2A was determined, and KAT2A restoration could completely rescue the impaired growth ability (left). Quantification of the colony formation assay results (right). **(E)** KAT2A deficiency effectively suppress the RCC subcutaneous tumor growth in nude mice (*n* = 5) versus the control group (left). The representative HE slides and matched intra-tumor Ki67 staining were shown (right). **(F)** The tumor volumes in two groups were measured in the indicated days, and the serial tumor growth curves were generated. **(G)** Representative pictures of two independent RCC organoids transfected with shRNAs targeting KAT2A or control lentivirus for 21 days. The quantification of organoid diameters (right panel) was shown as the means ± SD. **(H)** Quantification of organoid sizes of the PDOs. ***P < 0.001.

Given the significant associations between the KAT2A expression and prognosis of RCC in large patient samples, we intended to further figure out the functional role of KAT2A. Firstly, we inquired about the KAT2A expression levels from the Cancer Cell Line Encyclopedia (CCLE) dataset and selected two KAT2A^high^ cell lines (Caki-2 and A498) for the following studies, in which KAT2A was preferentially expressed (Supplementary Table 2). We then utilized the *CRISPR/Cas9* technology to knockout KAT2A and generate stable GFP-tagged KAT2A-overexpressing clones in two cell lines. The KAT2A-knockout efficiency was determined via Western blotting (Figure 2A and Supplementary Figure 2A). The CCK-8 assays revealed that KAT2A deficiency significantly inhibited the RCC growth compared with that in the WT control group, which was consistent in three independent cell lines (Figure 2B). Besides, KAT2A overexpression significantly promoted the RCC cell colony formation efficiency relative to the WT control cells based on the 3D soft-agar assays (Figure 2C). Additionally, KAT2A deficiency inhibited the RCC clone formation ability, whereas the restoration of KAT2A completely rescued the limited cell growth caused by KAT2A ablation (Figure 2D). Moreover, we also conducted the tumor xenograft studies to assess the oncogenic roles of KAT2A *in vivo.* We observed that KAT2A depletion significantly impeded the tumor growth relative to those in the control groups, which was compared by tumor weight, tumor volumes, and intratumor Ki-67 IHC staining scores (Figures 2E,F and Supplementary Figure 2B). Lastly, we also performed a RCC organoid model with organoids derived from two different RCC patients to further demonstrate the potent clinical significance of KAT2A. The lentivirus-mediated KAT2A knockdown significantly inhibited the RCC proliferation in two different RCC organoids, as quantified by the average organoid sizes (Figures 2G,H). Taken together, these data suggested that KAT2A could act as an oncogene that drives RCC proliferation *in vitro* and *in vivo*.

### Overexpressed KAT2A Accelerates RCC Metastatic Phenotype *in vitro* and *in vivo*

**FIGURE 3:**
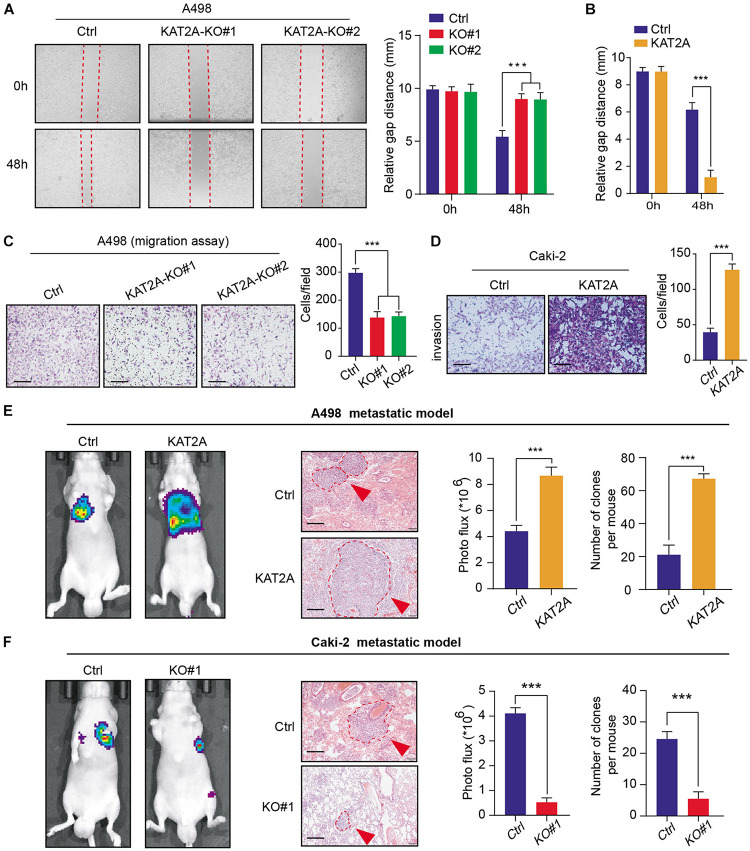
KAT2A enhances RCC metastatic ability *in vitro* and *in vivo*. **(A)** Wound healing assay revealed that KAT2A deficiency significantly inhibit the migration of A498 cells, **(B)** whereas KAT2A overexpression enhance the migrated ability of A498 cells compared with the control cells. **(C,D)** Cell migration and invasion experiments of A498 and Caki-2 cells following the knockout or overexpression of KAT2A. Representative pictures were selected (scale bars = 250 μm) and quantification of the cell migration and invasion assay results were shown on the right of each panel. **(E)** KAT2A overexpression remarkably enhanced the RCC lung metastasis in nude mice (*n* = 5). The representative pictures of bioluminescence imaging (BLI) of mice 8 weeks after portal vein injection of A498 cells with KAT2A overexpression or vector control cells (left). H&E-stained lung sections (scale bars = 250 μm) (middle). Quantification of the results of BLI signals and lung metastases (right). **(F)** Additionally, KAT2A deficiency significantly suppressed the distal metastasis of Caki-2 cells. The representative images of bioluminescence imaging (BLI) of mice 8 weeks after portal vein injection of Caki-2 cells with KAT2A ablation or vector control cells (left). H&E-stained lung sections (scale bars = 250 μm) (middle). Quantification of the results of BLI signals and lung metastases (right). ***P < 0.001.

Previous studies have already indicated that a high KAT2A is also correlated with advanced N stages. As a result, we intended to further investigate the roles of KAT2A in the RCC metastatic process. The wound-healing assay revealed that overexpressed KAT2A notably enhanced the migration speed of RCC cells, however, KAT2A deficiency remarkably inhibited their migratory efficiency (Figures 3A–C). Besides, forced expression of KAT2A also markedly increased the invasive ability of RCC Caki-2 cells based on the Transwell Matrigel invasion assay (Figure 3D). Additionally, *in vivo* assays were further conducted to assess the effects of KAT2A on RCC metastasis. The luciferase-labeled RCC cells with a modified KAT2A were established and injected into the tail vein of BALB/c nude mice. Then, we detected the bioluminescence signals with a regular interval to capture the metastatic RCC cells into the lung. After 8 weeks, we observed that KAT2 overexpression significantly promoted the ability of RCC cells to metastasize into the lung compared with the control group in the Caki-2 metastatic model as quantified by BLI signals and numbers of lung metastases, whereas KAT2 ablation significantly inhibited the distal metastasis in the A498 metastatic model (Figures 3E,F). Collectively, these findings supported the critical role of KAT2A in promoting RCC distal metastasis.

### Overexpressed KAT2A Alters the Oncogenic Transcriptome in RCC, Especially the Glycolytic Metabolism

**FIGURE 4:**
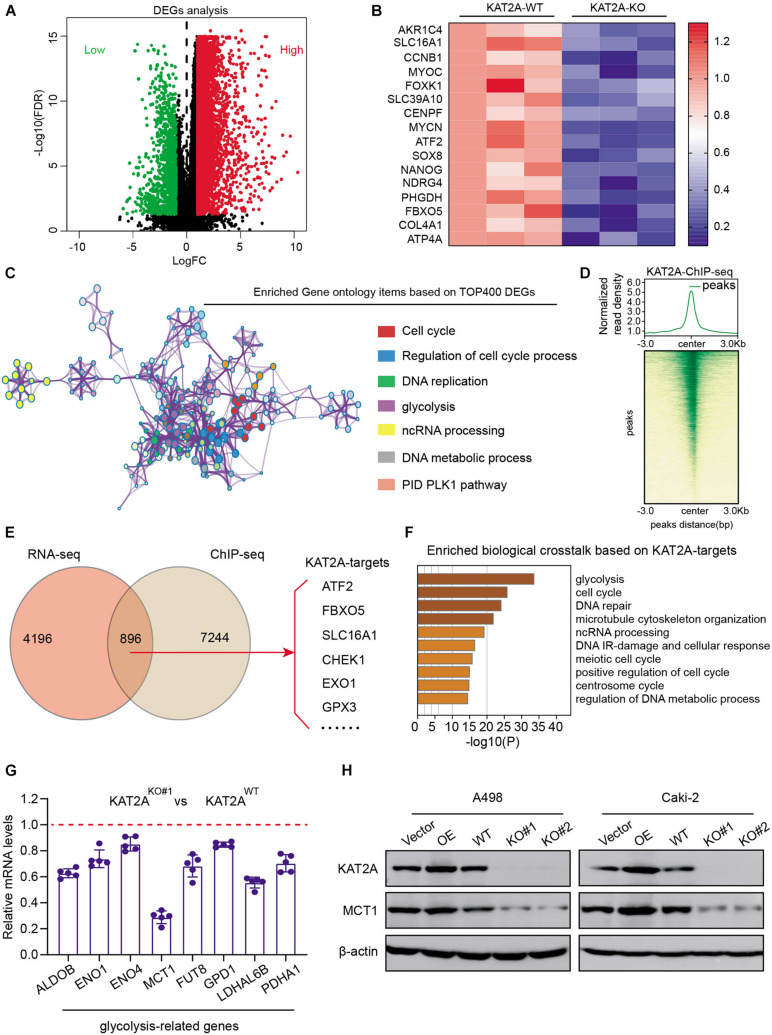
KAT2A alters oncogenic transcriptome and mainly activates downstream MCT1 expression. **(A)** Volcano plot revealing the differentially expressed genes (DEGs) between KAT2A deficiency and WT control Caki-2 cells. **(B)** Heatmap summarizing independent RNA-seq expression data of Top DEGs, as indicated. **(C)** Enriched Gene ontology (GO) items based on TOP400 DEGs were shown, where different colors represented different GO biological processes. **(D)** ChIP-Seq summary diagram of KAT2A-binding intensities across KAT2A peaks in Caki-2 cells (upper). Heatmap reflecting ChIP-seq signals displayed from −3 to + 3 kb surrounding the center of each peak (lower). **(E)** Venn diagram showing the overlapped genes with ChIP-seq data and DEGs. **(F)** Enriched biological crosstalk based on KAT2A-targets. **(G)** RT-qPCR analysis of indicated gene expressions in KAT2A-WT and KAT2A-deficient cells. **(H)** The associations between KAT2A and MCT1 protein levels were further confirmed via Western blotting assay in A498 and Caki-2 cells.

To further figure out the underlying mechanisms by which KAT2A enhances RCC growth and tumor progression, we performed the RNA-seq technology to compare the differential transcriptome between KAT2A-WT and KAT2A-KO Caki-2 cells. The differential analysis found that 5,092 differentially expressed genes (DEGs) were found with the cutoff of | Log(fold change)| > 1 and FDR < 0.01 (Figure 4A and Supplementary Table 3). The top oncogenic DEGs were further selected and illustrated by a heatmap (Figure 4B). We then performed the Gene ontology (GO) analysis to find that these DEGs were mainly enriched in cell cycle, DNA replication, glycolysis, and PID PLK1 pathway, in accordance with the oncogenic role of KAT2A in RCC (Figure 4C). To further narrow down the potential KAT2A downstream targets, we also conducted the chromatin immunoprecipitation sequencing (ChIP-seq) technology in Caki-2 cells using the anti-KAT2A antibody. The average occupancy of KAT2A peaks and the corresponding genome-wide ChIP-seq intensity plot confirmed the superior efficiency of enrichment (Figure 4D). We then mapped the KAT2A binding peaks to screen the nearest genes and overlapped them with the DEGs to identify a total of 896 genes, which were determined as the KAT2A-signature (Figure 4E and Supplementary Table 4). KEGG analysis of KAT2A-signature revealed that these genes were mainly enriched in glycolysis, cell cycle, DNA repair, and ncRNA processing crosstalk (Figure 4F). Given that the top hit of KAT2A-downstream crosstalk was glycolytic metabolism, we then concentrated on this pathway and screened the glycolysis-related genes among the KAT2A-signature. Subsequently, genes that were representative of the pivotal drivers associated with glycolysis were chosen, including ALDOB, ENO1, ENO4, MCT1, FUT8, GPD1, LDHAL6B, and PDHA1. Among these potent genes, mRNA expression levels of MCT1 decreased the most sharply upon KAT2A ablation compared with the other glycolytic candidates (Figure 4G). The Western blot assay further confirmed the roust associations between KAT2A and MCT1 expression levels, in which MCT1 protein levels elevated or reduced with KAT2A overexpression or deficiency (Figure 4H), respectively. Taken together, we utilized the high-throughput sequencing and validations to identify MCT1 as the vital downstream target of KAT2A for further analysis.

### KAT2A Mainly Activates and Depends on MCT1 to Maintain the Oncogenic Phenotype in RCC

**FIGURE 5:**
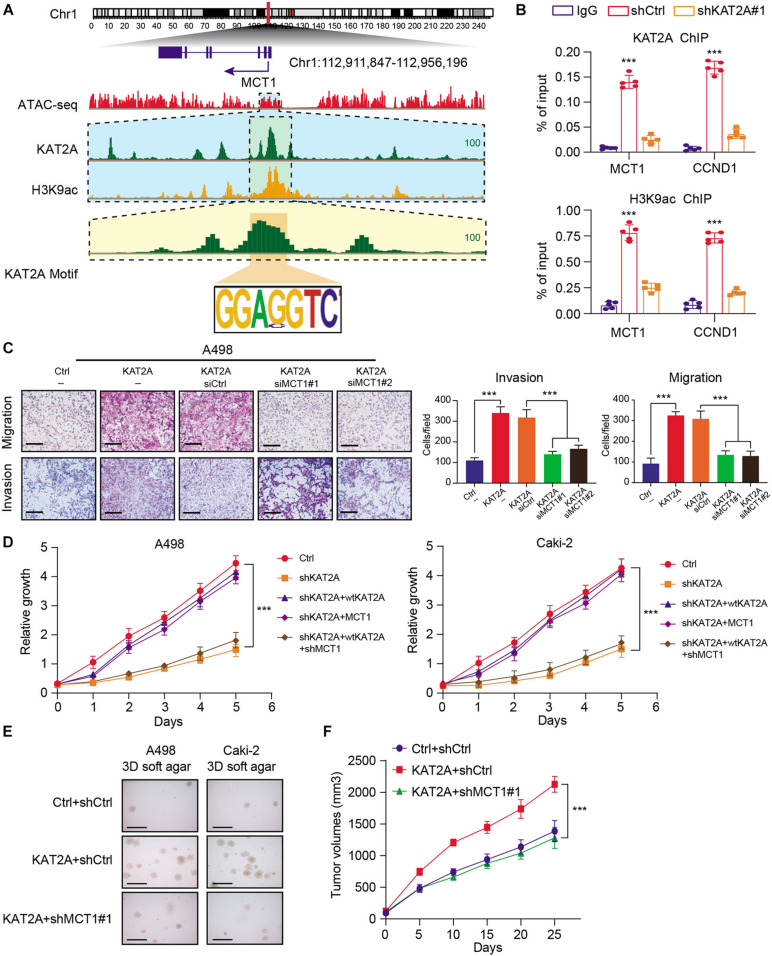
KAT2A enhances the H3K9ac abundance at MCT1 promoter and depended on MCT1 to accelerate RCC malignant progression. **(A)** IGV diagram indicating the multiple tracks of KAT2A and H3K9ac binding signals at the MCT1 promoter loci, as indicated. **(B)** ChIP-qPCR experiment of KAT2A binding and H3K9ac in genes, as indicated. **(C)** Representative pictures (scale bars = 250 μm, left) and quantification analysis (right) of cell migration and invasion abilities of KAT2A-overexpressed A498 cells transfected with the MCT1 siRNAs or controls. **(D)** CCK-8 assays of KAT2A knockdown Caki-2 and A498 cells underwent MCT1 overexpression or combined management of WT KAT2A restoration and scramble or MCT1 shRNA. **(E)** 3D soft-agar assays revealing that MCT1 knockdown could impair the growth ability of KAT2A-overexpressed RCC cells (A498 and Caki-2). **(F)** The tumor volume was assessed every other day, and tumor growth curves were generated. ***P < 0.001.

To further determine the specific epigenetic regulations of KAT2A on MCT1, we firstly focused on the aberrant histone modification markers associated with KAT2A. Previous studies have already indicated that acetylation of lysine 9 on histone 3 (H3K9ac) is an active marker through releasing paused RNA polymerase II to facilitate gene expressions. H3K9 acetylation is previously reported to be mainly mediated by KAT2A. We thus performed the ChIP-seq analysis using the H3K9ac antibody in Caki-2, parallelly. We integrated the ATAC-seq data of the RCC samples and the corresponding Chip-seq data to detect the significantly enriched peaks of KAT2A across MCT1. We observed the co-occupancy of KAT2A and H3K9ac at the promoter regions of MCT1 in the IGV plot, in accordance with our speculations (Figure 5A). Besides, ChIP-qPCR experiments further demonstrated that KAT2A loss significantly decreased H3K9ac levels at the MCT1 and CCND1 gene loci (Figure 5B). The notably positive association between KAT2A and MCT1 was further confirmed by Pearson analysis with *r* = 0.29 and *P* < 0.0001 (Supplementary Figure 3A). We also observed that patients with a high MCT1 were associated with a worse overall survival (OS) prognosis relative to a low MCT1 group (Supplementary Figure 3B). As expected, specific siRNAs were utilized to knock down the KAT2A expressions, which significantly inhibited the KAT2A-induced RCC migration and invasion (Figure 5C). In addition, MCT1 knockdown via lentivirus infection of specific shRNAs remarkably abrogated the effects of reconstituting wild-type KAT2A in RCC cells (Caki-2 and A498) in which KAT2A had been knocked out, indicating that KAT2A depended on MCT1 to promote RCC proliferation (Figure 5D). The 3D soft-agar clone formation assays further confirmed similar results (Figure 5E). Lastly, knockdown of MCT1 significantly impeded KAT2A-induced RCC growth in subcutaneous tumor models, as quantified by serial tumor volumes (Figure 5F). Collectively, these findings suggested that KAT2A enhances RCC malignant progression through upregulating the MCT1 expression.

### Targeting MCT1 (AZD3965) Has Clinical Significance to Suppress the Tumor Progression in KAT2A^high^ RCC

**FIGURE 6:**
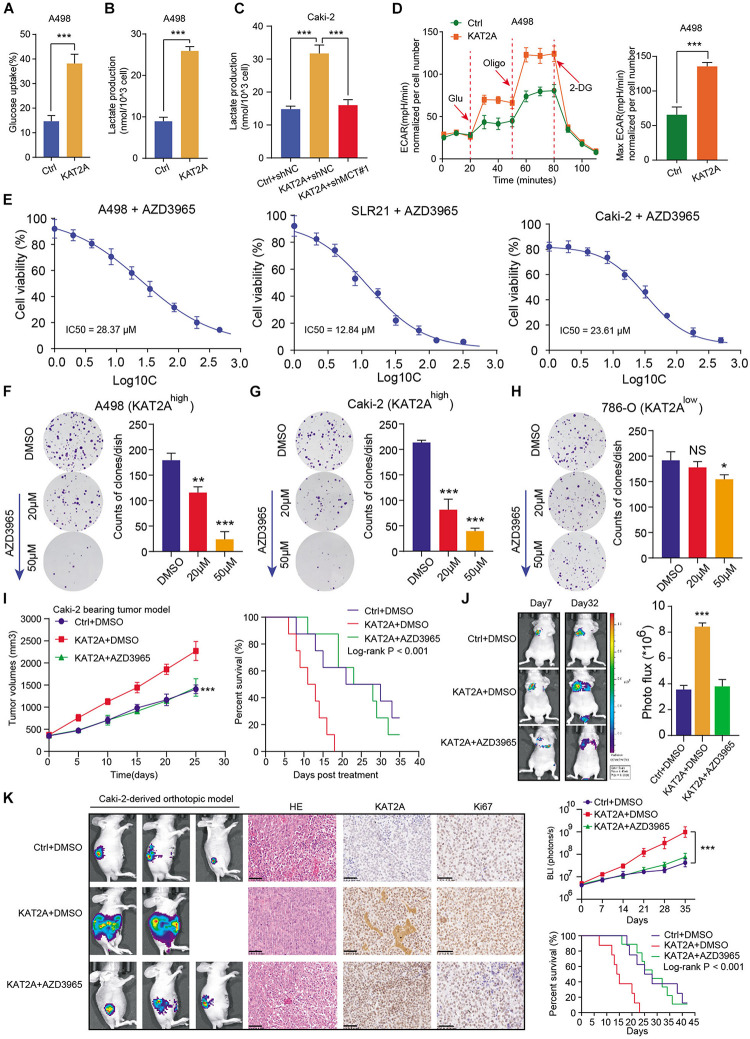
MCT1 inhibitor (AZD3965) was effective to suppress tumor growth of KAT2A^high^ RCC. **(A,B)** Overexpression of KAT2A induced glucose uptake and lactate production in A498 cells. **(C)** MCT1 knockdown inhibits lactate production in KAT2A-overexpressed Caki-2 cells. **(D)** The ECAR profile was assessed in KAT2A-overexpressing A498 with a Seahorse XF24 analyzer for 100 min. **(E)** Detection of IC50 of AZD3965 in three independent RCC cell lines (A498, SLR21 and Caki-2). **(F–H)** AZD3965 could significantly inhibit the colony formation in KAT2A^high^ cell lines (A498 and Caki-2) in a dose-dependent manner, but hardly work in 786-O. **(I)** Detection of tumor volumes and survival outcomes of mice in three groups, including Ctrl + DMSO, KAT2A + DMSO, and KAT2A + AZD3965. **(J)** AZD3965 could effectively inhibit the distal metastatic ability of KAT2A-overexpressing Caki-2 via tail vein injection, as indicated by Photo flux signals (left). **(K)** Representative pictures of Caki-2-derived renal orthotopic xenografts in three groups, as indicated in the left panel (*n* = 5 per group). Representative diagrams of H&E slides and matched staining scores of KAT2A and Ki67 (middle). Quantification of the serial photon flux signals in indicated tumor regions and prognostic difference of mice from three groups (right). *P < 0.05, **P < 0.01, and ***P < 0.001.

Given that the previous results have suggested that KAT2A could regulate the glycolysis crosstalk, we thus detected that the upregulation of KAT2A in Caki-2 cells significantly increased glucose uptake and lactate production, which could be notably suppressed upon MCT1 inhibition (Figures 6A–C). In addition, the extracellular acidification rate (ECAR) kinetic profiles further demonstrated that the glycolytic activity of A498 and Caki-2 cells could be notably suppressed with KAT2A knockout (Figure 6D and Supplementary Figure 3C). These data were in accordance with the fact that KAT2A promoted the glycolytic process depending on MCT1. Considering that there was a lack of available inhibitors targeting KAT2A, we thus speculated whether MCT1 inhibitor (AZD3965) could be effective to suppress KAT2A-induced RCC progression. First of all, we detected half of the maximal inhibitory concentration (IC50) values of AZD3965 in RCC cell lines (A498, SLR-21, and Caki-2) (Figure 6E). Based on the results from the pharmacological parameter assessment, we found that AZD3965 could significantly inhibit the *in vitro* growth of A498 and Caki-2 cells in a dose-dependent manner (Figures 6F,G). However, limited inhibitory efficiency of AZD3965 was observed in 786-O cells, as there might be low KAT2A/MCT1 expression levels (Figure 6H). Additionally, the *in vivo* assays further demonstrated that AZD3965 could significantly inhibit the increasing tumor volumes and distal lung metastases in KAT2A-overexpressing RCC (Figures 6I,J). Lastly, we further conducted the Caki-2-derived orthotopic ccRCC model *in vivo* and found that AZD3965 was notably effective in ccRCC tumors with overexpressed KAT2A to improve the overall prognosis of mice, which was quantified by serial bioluminescence signals and Kaplan-Meier analysis (Figure 6K). Taken together, we proposed that MCT1 inhibitor (AZD3965) was an effective strategy to treat KAT2A^high^ RCC, which has a clinical significance to be further investigated.

## Discussion

The morbidity and mortality of RCC has gradually increased yearly and the overall prognosis of RCC remains unsatisfactory (Powles et al., 2020; Braun et al., 2021; Jonasch et al., 2021). How to identify novel biomarkers and therapeutic targets are promising to optimize the current treatment of RCC. It has been well-known that loss-of-function mutations and abnormal expressions of epigenetic drivers could result in tumorigenesis and progression of RCC (Nargund et al., 2017; Zhang et al., 2021a,b). Apart from aberrant classical chromatin regulator genes like *VHL*, *PBRM1*, *BAP1*, and *SETD2*, the existence of other epigenetic vulnerability in RCC remains unclear. In this study, we utilized the bioinformatic methods to find that chromatin modifier KAT2A was significantly high in the tumor samples than normal tissues. KAT2A was demonstrated to function as an independent factor and correlate with the prognosis of RCC. Elevated KAT2A could promote RCC proliferation and metastasis *in vitro* and *in vivo*. High-throughput sequencing data indicated that KAT2A deficiency could downregulate a series of cancer-associated crosstalk, and the top hit was glycolysis. Low-throughput validations identified that MCT1 was the downstream target of KAT2A. Mechanistically, KAT2A could mediate the H3K9ac abundance at the promoter region of MCT1 to activate its expression levels. Rescue assays further verified that KAT2A mainly depended on MCT1 to drive the progression and distal metastasis of RCC. Based on these findings, we found that MCT1 inhibitor (AZD3965) could effectively inhibit the oncogenic phenotypes induced by KAT2A in RCC.

Increasing studies have already indicated that KAT2A was an epigenetic oncogene in various cancers, like pancreatic carcinoma, gastric carcinogenesis, and nasopharyngeal carcinoma (Sun et al., 2016; Bondy-Chorney et al., 2019; Wang et al., 2020). Given the previous results that a high KAT2A correlated with the clinical risk parameters and overall survival (OS) in RCC, no current studies uncovered the underlying mechanisms between KAT2A and oncogenic phenotypes in kidney cancer. Besides, KAT2A was reported to possess dual functions as a histone H3 succinyltransferase and acetyltransferase (Moris et al., 2018; Sandoz et al., 2019). KAT2A could promote leukemia stem-like cell propagation or regulate the tamoxifen resistance in breast cancer (Oh et al., 2020). However, little was investigated between KAT2A and tumor metabolism. We combined the transcriptome sequencing and ChIP sequencing to further narrow down the KAT2A-downstream targets. We found that nearly 20% of DEGs both have significantly altered expression levels and KAT2A binding peaks. We regarded that there might be lots of DEGs that were not regulated directly by KAT2A, and more technical repeats would be helpful to reduce the false positive rate. Through low-throughput validations, we demonstrated that MCT1 was the *bona fide* target of KAT2A. As well known, the solute carrier 16 (SLC16) family of genes mainly encode these MCTs, including MCT1/SLC16A1, MCT2/SLC16A7, MCT3/SLC16A8, and MCT4/SLC16A3 (Jones and Morris, 2016; Park et al., 2018). Moreover, MCT1, MCT2, and MCT4 are preferentially expressed in multiple cancer cells to facilitate lactate exchanges in tumor microenvironment. Targeting MCTs was proven to significantly suppress the growth or metastasis of solid tumors, like breast cancer, lung carcinoma, and glioblastoma (Payen et al., 2020; Sandforth et al., 2020). Inhibitors of MCT4 are in the discovery phase and AZD3965 (a selective inhibitor) is already in the pre-clinical trials to treat cancers (Dalton et al., 2021; Wang et al., 2021). Particularly, previous studies have already indicated that MCT1 was an unfavorable factor in RCC (Guo et al., 2019). However, no studies have indicated the causes for the high expression levels of MCT1 in RCC and assessed the pharmacological efficacy of MCT1 to suppress RCC. In this study, we clarified that KAT2A could activate the transcription of MCT1 via inducing abundance of H3K9ac at the promoter of MCT1, partially explaining the reasons for the aberrant levels of MCT1. Whether there existed other modifications like N6-methyladenosine (m6A), ubiquitination-mediated degradations or methylation that contribute to dysregulations of MCT1 remains intriguing to explore further. Considering that there were currently no available data on KAT2A inhibitors, we found that MCT1 inhibitor (AZD3965) could significantly inhibit the RCC growth induced by KAT2A.

However, there still existed several limitations in the current study. First of all, owing to the limited patients in our hospital, we were still uncertain to identify the exact cutoff to discriminate the high-KAT2A RCC samples. Secondly, apart from the proliferation and distal metastasis, whether KAT2A mediated other oncogenic processes, like stemness, angiogenesis, or drug resistance, was interesting to systematically elucidated. Furthermore, we mainly conducted the experimental assays in KAT2A-KO and KAT2A-OE cell models, without considering the genetic heterogeneity across cells. We should obtain more cell lines to assess the expression levels of KAT2A in RCC. The associations between KAT2A expression levels and AZD3965 drug sensitivity should be quantitatively assessed for pharmacological researches. How to define the cutoff to clarify the KAT2A^high^ and KAT2A^low^ RCC was meaningful to guide the management of AZD3965. Large pre-clinical trials and patient-derived xenografts (PDX) were warranted to assess the potential differences of inhibitory efficacy of AZD3965 in the KAT2A^high^ and KAT2A^low^ RCC samples.

## Conclusion

Taken together, these findings derived from the TCGA analysis, functional validations, and high-throughput sequencing data highlighted that KAT2A was an independent biomarker and a potentially therapeutic target in RCC. KAT2A depended on MCT1 to drive malignant phenotypes. and AZD3965 was significantly effective to suppress the tumor progression induced by KAT2A. How to reasonably define the KAT2A^high^ and KAT2A^low^ RCC and assess the pharmacological security of AZD3965 will be of great importance to deal with in following researches.

## Data Availability Statement

The data presented in the study are deposited in the Sequence Read Archive (SRA) data repository, accession number (PRJNA732761).

## Ethics Statement

The studies involving human participants were reviewed and approved by the Ethics Committee of the first affiliated hospital of Bengbu Medical College. The patients/participants provided their written informed consent to participate in this study. The animal study was reviewed and approved by the Ethics Committee of the first affiliated hospital of Bengbu Medical College.

## Author Contributions

LC conceived the concept of this research. YG, BL, and YL performed the experimental research and data analysis. WS, WG, and SM collected the samples and data analysis. LC and YG wrote and reviewed the draft of the manuscript. All authors approved the final manuscript.

## Conflict of Interest

The authors declare that the research was conducted in the absence of any commercial or financial relationships that could be construed as a potential conflict of interest.

## Footnotes

1 https://portal.gdc.cancer.gov/

2 https://dcc.icgc.org/

3 https://www.ncbi.nlm.nih.gov/geo/query/acc.cgi?acc=GSE40435

4 https://gdc.cancer.gov/about-data/publications/ATACseq-AWG

## Supplementary Material

Supplementary Figure 1 KAT2A was a risk factor in RCC. **(A)** KAT2A expression levels correlated with higher tumor grades and T stages. **(B)** The ROC curve indicating the predictive efficiency of KAT2A in the TCGA-KIRC cohort.

Supplementary Figure 2 KAT2A promoted the tumor growth of RCC. **(A)** The protein levels of KAT2A in Caki-2 cells with KAT2A overexpression or deficiency were validated via Western blotting. **(B)** KAT2A deficiency significantly inhibited tumor weight.

Supplementary Figure 3 KAT2A correlated with its downstream target of MCT1. **(A)** The expression levels of KAT2A correlated positively with MCT1 in the TCGA-KIRC cohort. **(B)** Kaplan-Meier analysis suggested that patients with a high MCT1 suffered from worse overall survival (OS) outcomes compared with those with a low MCT1 (Log-rank test *P* < 0.001). **(C)** The extracellular acidification rate (ECAR) kinetic profiles indicated that the glycolytic activity of Caki-2 cells could be significantly impaired with KAT2A deficiency.
