# Supplementary figures and images for: Oncogenic Chromatin Modifier KAT2A Activates MCT1 to Drive the Glycolytic Process and Tumor Progression in Renal Cell Carcinoma
Source: Front Cell Dev Biol. 2021 Jun 29;9:690796. doi: 10.3389/fcell.2021.690796 (PMC8276638; doi:10.3389/fcell.2021.690796)

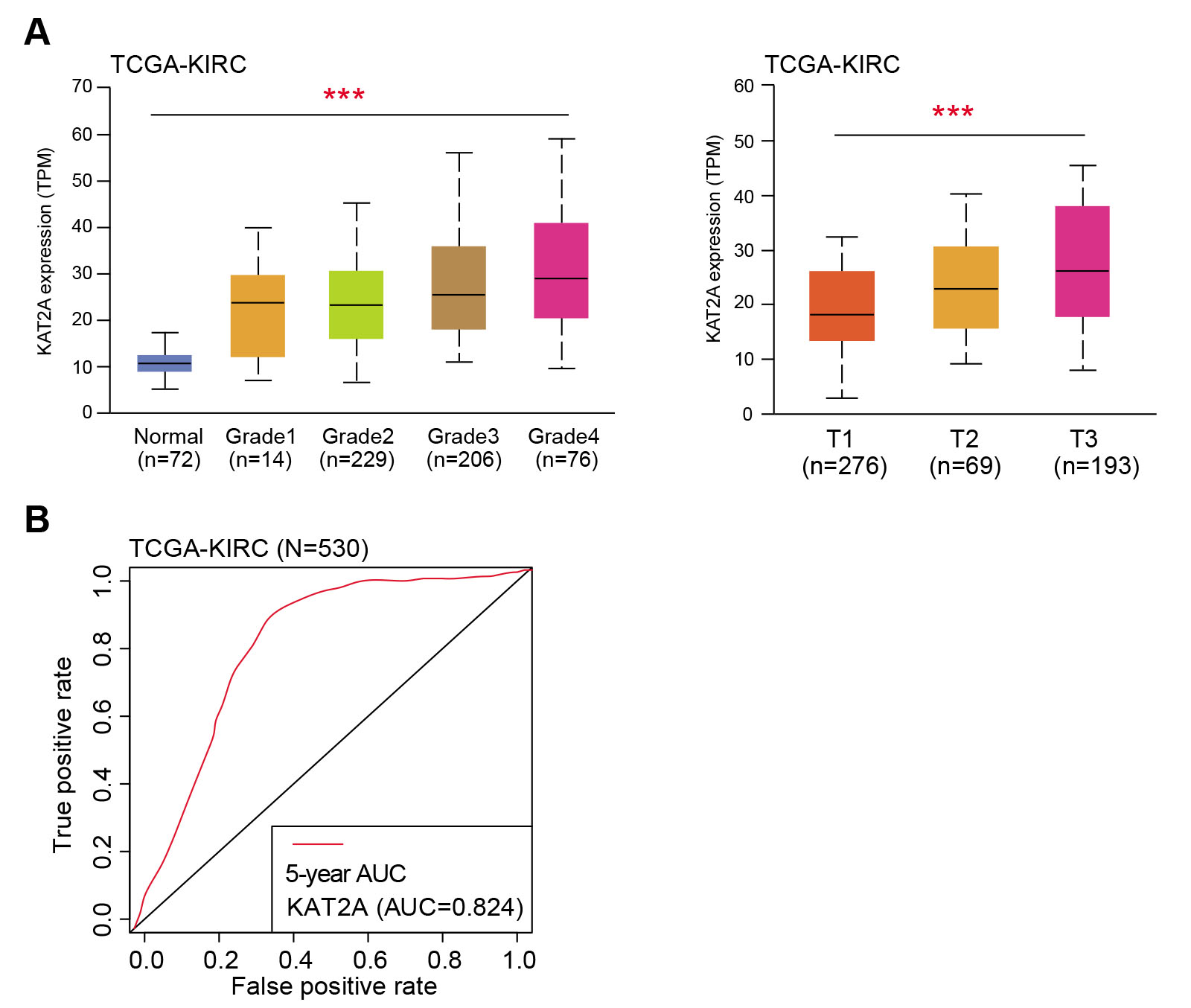

Supplement: Supplementary Figure 1 — KAT2A was a risk factor in RCC. (A) KAT2A expression levels correlated with higher tumor grades and T stages. (B) The ROC curve indicating the predictive efficiency of KAT2A in the TCGA-KIRC cohort. [file Image_1.JPEG]

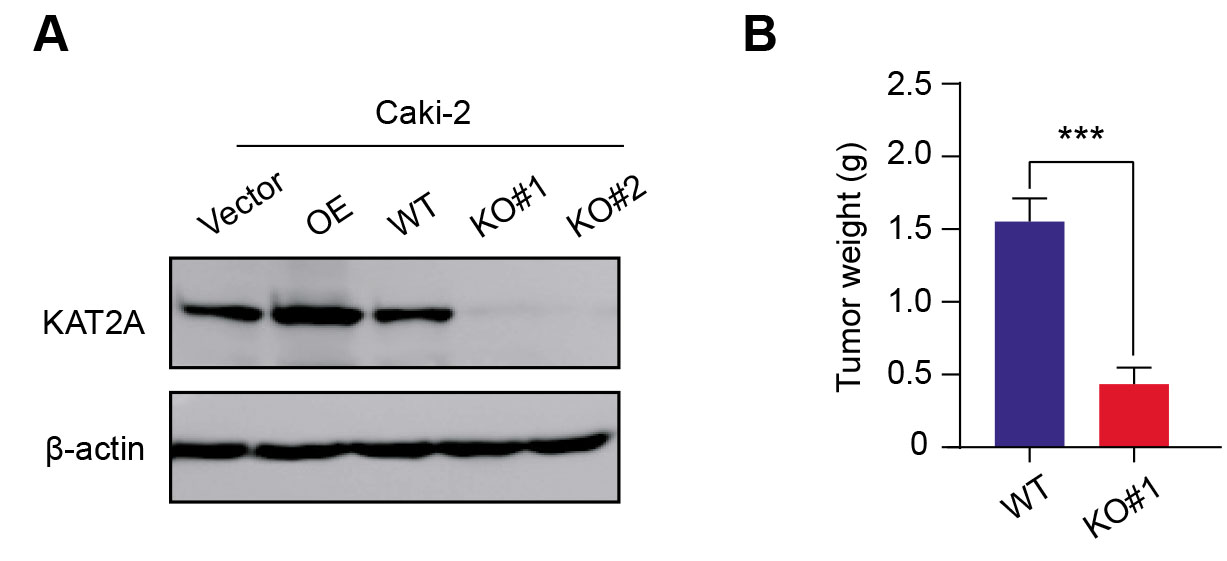

Supplement: Supplementary Figure 2 — KAT2A promoted the tumor growth of RCC. (A) The protein levels of KAT2A in Caki-2 cells with KAT2A overexpression or deficiency were validated via Western blotting. (B) KAT2A deficiency significantly inhibited tumor weight. [file Image_2.JPEG]

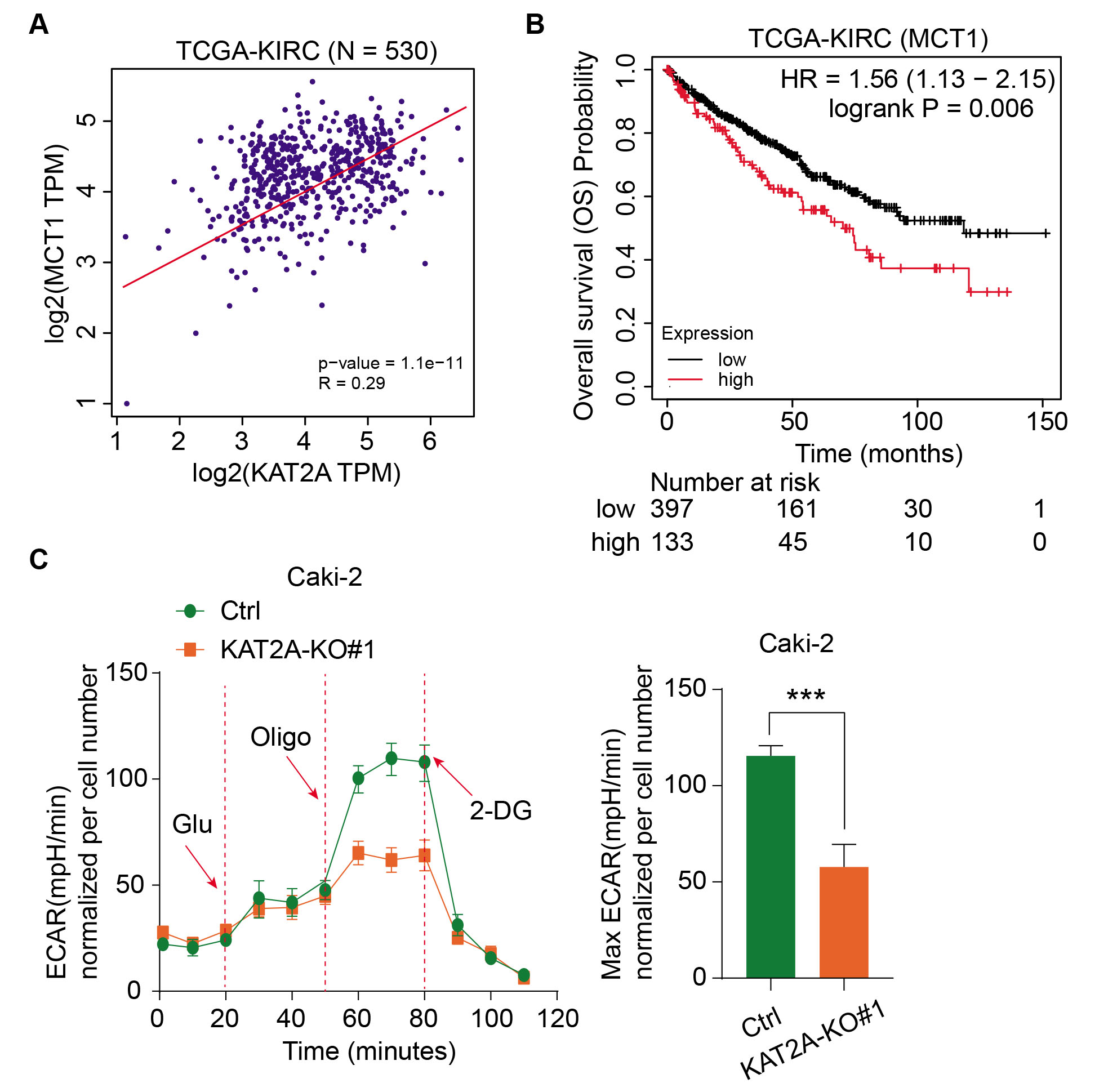

Supplement: Supplementary Figure 3 — KAT2A correlated with its downstream target of MCT1. (A) The expression levels of KAT2A correlated positively with MCT1 in the TCGA-KIRC cohort. (B) Kaplan-Meier analysis suggested that patients with a high MCT1 suffered from worse overall survival (OS) outcomes compared with those with a low MCT1 (Log-rank test P < 0.001). (C) The extracellular acidification rate (ECAR) kinetic profiles indicated that the glycolytic activity of Caki-2 cells could be significantly impaired with KAT2A deficiency. [file Image_3.JPEG]
